# The Impact of the Second Wave of the COVID-19 Pandemic on Non-COVID Hospital Care in a Tertiary Hospital in Spain

**DOI:** 10.3390/jcm12175507

**Published:** 2023-08-24

**Authors:** Antonia Gasch-Illescas, María Andrade-Arroyo, Antonio J. Vallejo-Vaz, Juan M. Praena-Fernández, José A. Guerrero, Enrique J. Calderón, Marina Pollán, Francisco J. Medrano

**Affiliations:** 1 Service de Santé Étudiants, Campus Arras, University of Artois, 62000 Arras, France; agillescas@gmail.com; 2 Escuela Internacional de Doctorado, University of Seville, 41004 Seville, Spain; 3 Infectious and Immune System Diseases, Epidemiology and Public Health, Instituto de Biomedicina de Sevilla, IBIS, Hospital Universitario Virgen del Rocío, CSIC, University of Seville, 41013 Seville, Spain; 4 Servicio de Medicina Interna, Hospital Universitario Virgen del Rocío, 41013 Sevilla, Spain; andradearroyomaria96@gmail.com; 5 Department of Medicine, Faculty of Medicine, University of Seville, 41013 Seville, Spain; ajvallejo-ibis@us.es; 6 Clinical Epidemiology and Vascular Risk, Instituto de Biomedicina de Sevilla, IBIS, Hospital Universitario Virgen del Rocío, Consejo Superior de Investigaciones Científicas, CSIC, University of Seville, 41013 Seville, Spain; sandube@cica.es; 7 Department of Statistics and Operations Research, University of Granada, 18071 Granada, Spain; jmpraenaf@gmail.com; 8 Servicio de Documentación Clínica, Hospital Universitario Virgen del Rocío, 41013 Seville, Spain; jantonio.guerrero.sspa@juntadeandalucia.es; 9 Instituto de Biomedicina de Sevilla, IBIS, HUVR, Junta de Andalucía, Consejo Superior de Investigaciones Científicas, CSIC, University of Seville, 41013 Seville, Spain; 10 Centro de Investigación Biomédica en Red de Epidemiología y Salud Pública (CIBERESP), Instituto de Salud Carlos III, 28029 Madrid, Spain; mpollan@isciii.es; 11 National Center for Epidemiology, Instituto de Salud Carlos III ES, 28029 Madrid, Spain

**Keywords:** COVID-19/epidemiology, pandemics, inpatients, Spain/epidemiology, hospitalization, hospital mortality, retrospective studies, humans, male, hospital care

## Abstract

In 2020, Spain ranked fourth among European countries with the highest excess mortality due to COVID-19 disease. This study evaluates the impact of the COVID-19 pandemic on non-COVID patients in a tertiary hospital during the second pandemic wave in Spain (22 June 2020–6 December 2020). Data from Virgen del Rocío University Hospital in Seville during that timeframe were compared with the data from the same period in the preceding two years (2018–2019). Between-group comparisons were performed using the Chi-squared test, Student’s *t*-test, or Mann–Whitney U tests, as appropriate. A total of 63,137 non-COVID patients were included in this study. During the second pandemic wave, a 19% decrease was observed in the annual number of non-COVID admissions overall (18,260 vs. 22,439, *p* < 0.001), but a 10% increase in the proportion of emergency admissions (60.6% vs. 54.93%, *p* < 0.001), a higher severity level of patients (1.79 vs. 1.72, *p* < 0.001), a longer in-hospital stay (7.02 vs. 6.74 days, *p* < 0.001), a 26% increase in non-COVID mortality (4.9% vs. 3.9%, *p* < 0.001), and a 50% increase in global mortality (5.9 vs. 3.9, *p* < 0.001) were also observed. In terms of both medical and surgical diagnoses, a significant reduction in the number of admissions and an increase in in-hospital mortality were observed. These results demonstrate the significant impact of the pandemic on hospital care, similar to what was previously observed during the initial wave in the same hospital. Our findings emphasize the need to include non-COVID patients when assessing the broad impact of the pandemic on healthcare, beyond its direct effects on COVID-19 patients.

## 1. Introduction

The causative agent of COVID-19 is the severe acute respiratory syndrome coronavirus 2 (SARS-CoV-2), a virus of the *Coronaviridae* family whose genetic sequence was published in February 2020 [1]. Since the start of the pandemic, SARS-CoV-2 has spread widely across the globe, with more than 767 million confirmed cases by June 2023 and more than 6.9 million deaths worldwide, according to the World Health Organization (WHO) [2].

Spain was the fourth country with the highest excess mortality in Europe in 2020 (+18.2% compared with the average mortality of the previous three years), closely similar to that observed in Slovenia (+18.4%) and Poland (+18.7%), and lower than the excess mortality registered in Liechtenstein (+21.5%) [3]. This worrying ranking at the European level was mainly driven by the high incidence of SARS-CoV-2 infection during the first pandemic wave in Spain, which caused an excess mortality of 21.7% in the first half of 2020 [3]. In the second half of 2020, however, the excess mortality in Spain stood at 14.7%, somewhat below the European average (+17.5%) and countries such as Portugal, Italy, Austria, Belgium, Slovenia, Poland, and Liechtenstein. The latter three countries suffered the highest excess mortality due to the pandemic in 2020, which was concentrated almost exclusively in the second half of 2020. The only country in Europe with no excess mortality in 2020 was Norway (−0.2%) [3]. The first doses of COVID-19 vaccines started to be administered on 14 December 2020 in the United States and on 27 December 2020 in the European Union [4]. The first doses in Andalusia—from where the data for the present study is taken—were administered on 28 December 2020 [5].

Studies published to date assessing the overall impact of the COVID-19 pandemic on non-COVID conditions and non-COVID mortality in hospital settings are still scarce. Studies generally point to a reduction in both medical and surgical hospital admissions, an increase in emergency admissions, a change in the profile of patients admitted to hospitals, and an increase in hospital mortality, when compared to pre-pandemic years [6,7,8]. A large study conducted in the USA between March 2020 and June 2021 shows an immediate decrease in the incidence of the most major inpatient diagnoses from March 2020. Hospitalizations for conditions such as neoplasms, hypertension, and diabetes returned to pre-pandemic levels in late 2020 and early 2021, while others, such as respiratory infectious diseases, did not return to pre-pandemic levels during this period [9]. Some studies show, however, a decrease in non-COVID in-hospital mortality during the pandemic, despite the admitted patients having a higher severity rate at the time of hospitalization [10].

As a consequence of the impact of the pandemic, healthcare services have been weakened around the globe, not only by the need to respond to the high burden posed by the care for COVID-19 patients but also by the difficulty of ensuring continued access to other basic healthcare services. Moreover, it is assumed that many patients did not seek medical care due to uncertainty and public health restrictions at the onset of the pandemic [11]. Face-to-face healthcare was provided almost exclusively for serious health problems. Hospital admissions and procedures decreased during this phase, e.g., elective surgeries became less common. However, the rate of intensive care unit (ICU) admissions increased, mainly due to the complications of COVID-19 patients requiring intensive care [12]. In response to the increased demand, hospitals adopted extraordinary measures to cope with the overflow, reallocating resources, creating new COVID areas in the hospital, reorganizing patient and professional flow protocols, etc. These changes also affected patients without COVID-19, further increasing the clinical impact, mainly in older people and those with comorbidities [13].

The purpose of this study is to assess the impact of the SARS-CoV-2 pandemic on the healthcare of the Virgen del Rocío University Hospital in Seville, Spain, during the second pandemic wave in Spain (from 22 June 2020 to 6 December 2020), in particular on the profile of patients hospitalized with non-COVID conditions and on non-COVID mortality.

## 2. Materials and Methods

### 2.1. Setting and Context

This research was carried out at the Virgen del Rocío University Hospital (VRUH), a 1500-bed tertiary care university hospital in Seville, Andalusia, Spain. This hospital is the reference one for a population exceeding 550,000 residents in Seville, offering both outpatient and inpatient services across various medical (including internal medicine and specialties) and surgical areas, and contributes around 10% of the total intensive care inpatient bed capacity within the Andalusia region (southern Spain). Additionally, the hospital provides services in accident and emergency care, critical care, mental health, pediatrics, obstetrics, and gynecology. In the year 2019, the hospital reported 48,765 hospital admissions, 42,854 surgical procedures, and 330,142 emergency admissions. [6]

The initial case of SARS-CoV-2 infection in the Andalusia region was identified in Seville on 26 February 2020. The Spanish government enforced the first lockdown measures on 15 March 2020, which included a requirement for non-essential workers to stay at home. This initial lockdown was in effect until 21 June 2020. Formal restrictions concerning access to healthcare services were not implemented at the regional level by authorities. Nevertheless, a contingency plan was put into action at the VRUH starting on 16 March 2020. This plan encompassed several measures: Healthcare professionals were encouraged to work remotely when physical presence was not necessary, both in primary and hospital care; outpatient consultations were prioritized through telephone and telemedicine in both primary and hospital care settings; non-essential face-to-face appointments were postponed in both primary and hospital care; scheduled elective surgeries were deferred, except for cases involving cancer and other conditions with high risks of clinical deterioration; urgent surgeries were maintained; the number of major ambulatory surgical procedures was reduced to 50%; pediatric and adult kidney transplants were suspended; code 0 liver and heart transplantations were continued; and the non-heart-beating organ donation program was suspended. These measures remained in place until the end of May 2020, when the start of a gradual de-escalation process began [6].

The local de-escalation measures that were applied in our center as of 25 May 2020 were the following: (i) the progressive incorporation of face-to-face visits for new outpatients, maintaining whenever possible the follow-up consultations via telemedicine; and (ii) the progressive restart of scheduled and elective surgical interventions and non-urgent invasive procedures, ruling out pre-procedural SARS-CoV-2 infection through the screening and detection of the virus via PCR in respiratory samples.

### 2.2. Study Periods

All the patients hospitalized at VRUH for any cause during the two periods described below were included in this study and compared with those admitted during the same periods in 2018 and 2019.

Period 1 (the second pandemic wave), from 22 June to 6 December 2020, corresponds to the second pandemic wave in Spain according to the National Epidemiological Surveillance Network (Red Nacional de Vigilancia Epidemiológica, RENAVE) of the Ministry of Health [14].

Period 2 (excess mortality during the second pandemic wave) involves three sub-periods (23 June 2020 to 26 June 2020, 12 July 2020 to 14 August 2020, and 6 September 2020 to 6 December 2020) corresponding to the three sub-periods of excess mortality for Andalusia during the second pandemic wave, according to the RENAVE [15].

The intervention measure implemented at the local level that had more impact on the course of the pandemic and the management of patients in the hospital was the start of the de-escalation of the first contingency plan (25 May 2020), which allowed the partial recovery of non-COVID healthcare activity.

### 2.3. Data Collection and Variables

Inpatient information was collected from the local Minimum Basic Data Set (MBDS), a standard management database and tool within the Spanish National Health System used for recording hospital admissions, as well as coding diagnoses and procedures. Diagnoses upon hospital discharge were encoded according to the Tenth Version of the International Classification of Diseases and Related Health Problems (ICD-10-EN) [16]. These diagnoses were further categorized into the refined version (APR, “all-patient refined”) of diagnostic-related groups (DRGs) based on the primary admission diagnosis from version 36.0 [17].

The data encompassed demographic details (age, sex, place of residence, and nationality); the variables linked to the hospitalization episode (the number of admissions during the study period, admission type, the length of stay (LOS), ICU days if applicable, APR-DRG severity level (indicating the predicted severity adjusted for age and comorbidity, ranging from 1 to 4 with 4 as the most severe), APR-DRG mortality level (indicating the predicted mortality adjusted for age and comorbidity, values from 1 to 4, with 4 as the highest predicted mortality)); the weighted value of each APR-DRG; the LOS index; diagnoses at discharge; and in-hospital mortality. The population served by the VRUH was recorded for each calendar year. Patient records were retrieved from the MBDS in an anonymous form.

### 2.4. Data Analysis

Quantitative data are presented as mean ± standard deviation (SD) and categorical variables and proportions using absolute counts (N) and relative frequencies (%). Comparisons between the studied groups were performed using Student’s *t*-tests or Mann–Whitney U tests for quantitative data, as appropriate (normally and non-normally distributed variables, respectively), and the Chi-square test (Fisher’s exact test where appropriate) for categorical data. In-hospital mortality rates were estimated. The analysis was carried out using IBM SPSS statistical software (IBM Corporation, Somers, NY, USA), version 26.0. Two-sided tests were conducted, and a significance level of *p* < 0.05 was established.

## 3. Results

### 3.1. Number of Hospitalizations

A total of 63,137 non-COVID patients (22,378 in 2018; 22,499 in 2019; and 18,260 in 2020) were admitted to the hospital during Period 1 (the second pandemic wave, from 22 June 2020 to 6 December 2020) and were included in this study. The mean annual number of patients in this period in 2018–2019 was 22,439, compared with 18,260 in 2020. These figures indicate a decrease of 18.6% in non-COVID admissions during the second pandemic wave in 2020 compared with the same period in the two pre-pandemic years (*p* < 0.001) (Table 1).

During Period 2 of the study (the addition of excess mortality periods identified during the second pandemic wave), 11,760 non-COVID patients were admitted in 2020, compared with an average of 15,146 non-COVID annual admissions in the same periods of 2018–2019, representing a 22.36% decrease in admissions in 2020 compared with the same period of the previous two years (*p* < 0.001).

### 3.2. Characteristics of Hospitalized Patients

Table 1 presents the characteristics of non-COVID patients hospitalized in Periods 1 and 2 of 2020, as well as during the corresponding dates in 2018–2019. In Period 1, the average age of the patients admitted in 2020 was slightly higher than that in the preceding two years (50.10 vs. 48.77 years, *p* < 0.001). Emergency admissions experienced a 10.3% rise in 2020 compared with 2018–2019 (60.6% vs. 54.9%, *p* < 0.001). Additionally, the average LOS increased by 4.15% in 2020 (mean 7.02 days, compared with 6.74 days in 2018–2019, *p* = 0.008).

The percentage of medical diagnoses rose by 1.65% in 2020 compared with the previous two years (60.0% vs. 58.7%, *p* = 0.004). This increase correlated with lower complexity of cases, as indicated by the mean APR-DRG weight (0.92 in 2020 vs. 1.17 in 2018–2019, *p* < 0.001), higher levels of severity (1.79 vs. 1.72, respectively, *p* < 0.001), and elevated mortality rates (1.51 vs. 1.44, *p* < 0.001). The proportion of male patients decreased by one percentage point compared with the pre-pandemic period (*p* = 0.008).

Similar, or even more pronounced differences, between these years were observed for Period 2 when comparing 2020 with the two previous years (Table 1).

### 3.3. Mortality

In Period 1, the overall in-hospital mortality rate in 2020 stood at 5.9 per 100 admissions (16.9% for COVID-19-diagnosed patients and 4.9% for non-COVID patients). Correspondingly, for the same period in 2018–2019, the in-hospital mortality rate for non-COVID cases was 3.9% (Table 2).

**Figure 1:**
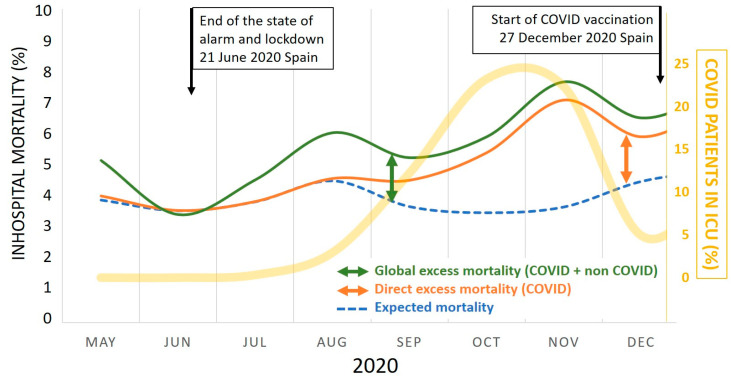
Expected and observed trends of in-hospital mortality at the VRUH between 22 June and 6 December 2020 (the second COVID-19 pandemic wave in Andalusia). VRUH, Virgen del Rocío University Hospital; ICU, intensive care unit.

These findings represent a 26% increase (*p* < 0.001) in mortality from causes unrelated to COVID-19 during the second wave of the pandemic when compared to previous years (2018–2019). The anticipated trends, based on the data from the years 2018–2019, and the observed in-hospital mortality trends during this second wave of the pandemic are illustrated in Figure 1.

**Figure 2:**
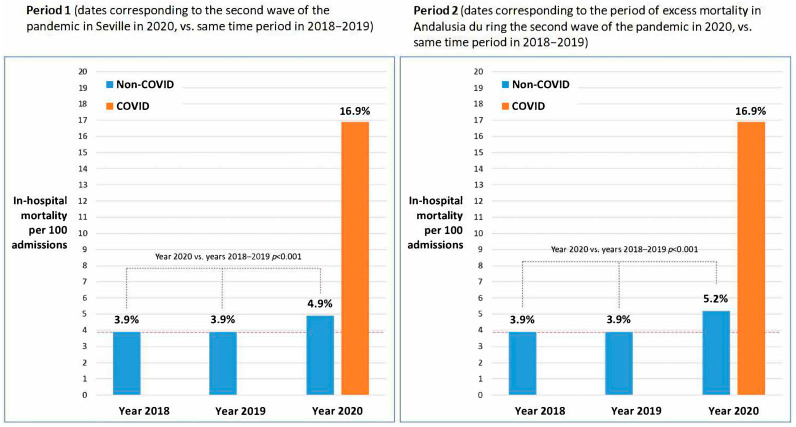
In-hospital mortality at VRUH during Periods 1 and 2 in 2020, compared with the same periods in 2018–19.

In Period 2, the overall hospital mortality rate was 6.3 per 100 admissions. Comparing Period 2 with Period 1, the hospital mortality rate remained relatively steady for patients diagnosed with COVID-19 (16.9 per 100 admissions), while experiencing a slight increase for non-COVID conditions (5.2 vs. 4.9 per 100 admissions), as depicted in Figure 2. An evident 33% rise (*p* < 0.001) in non-COVID mortality was observed during Period 2 in the year 2020 as compared to 2018–2019 (Table 1).

### 3.4. Changes in Non-COVID Patient Characteristics According to APR-DRGs

The demographic and clinical attributes of non-COVID patients hospitalized during the second wave of the pandemic for the 40 most frequent medical and surgical APR-DRGs are summarized in Tables S1 and S2, respectively, as compared to the corresponding period in 2018–19. Overall, across both medical and surgical admissions, there was an increase in the mean age, inpatient LOS in days, diagnosis severity, and in-hospital mortality rates in 2020 when compared with 2018–19.

Notably, non-COVID in-hospital mortality exhibited a 17% increase in 2020, in contrast to 2018–19, for medical conditions (7.01% vs. 5.97%, *p* < 0.001), and a 27% increase for surgical procedures (1.18% vs. 0.93%, *p* = 0.001).

Regarding medical diagnoses (Table S1), there was a significant decrease of 18% in medical admissions during the second pandemic wave (2020) compared with the same period in the previous two years. This reduction was even greater for surgical admissions (22%) (Table S2).

The individual medical diagnoses with a significant reduction in admissions (reduction ranging from 24% to 68%) were as follows (from the lowest to the largest reduction): “other digestive system diagnoses”; “other kidney and urinary tract diagnoses, signs and symptoms”; “antepartum without surgical procedure”; “other gastroenteritis, nausea, and vomiting”; “other disorders of the nervous system”; “other ear, nose, mouth, throat, and cranial or facial diagnoses”; “other skin, subcutaneous tissue, and breast disorders”; “other esophageal disorders”; “cardiac catheterization for other non-coronary conditions”; “other disorders of the liver”; “ infections of upper respiratory tract”; and “other chemotherapy”. The only diagnosis for which an increase in admissions was observed (77%) was “lymphoma, myeloma, and non-acute leukemia”. In-hospital mortality increased significantly for two medical diagnoses: “acute kidney injury” and “septicemia and disseminated infections”. The detailed results for these and other variables specific to non-COVID patients with medical diagnoses are shown in Table S1.

Regarding surgical diagnoses (Table S2), we observed a significant decrease in the number of admissions in 2020 (ranging from 27% to 79%) compared with the previous years in 15 of the most frequent diagnoses (from the lowest to the largest reduction): “percutaneous cardiac interventions without AMI”; “urethral and transurethral procedures”; “other ears, nose, mouth, and throat procedures”; “skin graft for skin and subcutaneous tissue diagnoses”; “mastectomy procedures”; “ major large bowel procedures”; “ hernia procedures except inguinal, femoral, and umbilical”; “foot and toe procedures”; “cholecystectomy”; “breast procedures except mastectomy”; “other significant hip and femur surgeries”; “anal and perineal procedures”; “ inguinal, femoral and umbilical hernia procedures”; “knee joint replacement”; and “tonsil and adenoid procedures”. In-hospital mortality increased significantly for two surgical procedures: “cardiac valve procedures without AMI or complex principal diagnoses” (more than five-fold increase) and “moderately extensive O.R. procedure unrelated to principal diagnosis” (almost six-fold increase). The detailed results for these and other variables specific to patients with surgical diagnoses are shown in Table S2.

## 4. Discussion

This study offers insights into the broader effect of the second wave of the COVID-19 pandemic on healthcare delivery within a tertiary university hospital, beyond the direct effect of SARS-CoV-2 infection, substantially affecting the care and outcomes of patients not affected by COVID-19.

Our research encompassed admission figures, demographic and clinical characteristics, and mortality rates for patients hospitalized during the local pandemic’s second wave, which spanned roughly the latter half of 2020. This comprehensive analysis unveiled shifts in the clinical profile and treatment of patients who were not directly impacted by COVID-19 but experienced indirect consequences of the pandemic.

Among the findings, this study highlights a reduction in hospital admissions, a higher clinical complexity of non-COVID inpatients, and a rise in in-hospital mortality during the second pandemic wave, in contrast with the pre-pandemic years.

In terms of admissions, during the second pandemic wave, the cumulative incidence of hospitalizations decreased by 18.6% compared with the previous two years. This reduction is even greater in the period of excess mortality (22.3% decrease compared with the previous two years). This large drop in admissions is similar to that observed in a prior article analyzing the number of hospitalizations in 201 hospitals in 36 US states after the declaration of the pandemic in 2020, compared with the previous year [18]. Another study in Ethiopia, comparing admissions and the number of non-COVID clinic visits in a tertiary hospital, also concluded that there was a clear decrease in the number of inpatients during the pandemic [11].

During the second pandemic wave in the VRUH, only admissions due to “lymphoma, myeloma, and non-acute leukemia” increased significantly, as was also observed during the first wave [6]. This can be attributed to the absence of local restrictions for malignant disorders or conditions with a risk of fast clinical deterioration. Additionally, the significant reduction in outpatient oncology treatments led to an influx of these patients being admitted to the hospital for therapy.

Conversely, there was a discernible shift in the profile of hospitalized patients during both study periods when contrasted with previous years. This change may have been due to both the mobility restrictions implemented and the patients’ concerns about getting infected with SARS-CoV-2, which may have resulted in longer delays in seeking healthcare and an increase in the severity and complexity of patient conditions. In our study, during the second pandemic wave, the mean age of patients increased slightly by 1.33 years compared with the mean of the previous two years. More importantly, there was an increase in emergency admissions, LOS, severity, complexity, and mortality level of patients, whereas the proportion of males decreased. As for the second period analyzed, specific to the officially identified excess mortality sub-periods within the second wave, the mean age of the hospitalized patients also increased but to a lesser extent than in the first period. The rest of the characteristics followed the same pattern, with an increase in in-hospital mortality, compared with previous years, being even more noticeable (5.2% vs. 3.9%). These data are similar to those observed in a study in Melbourne, which showed a change in patient characteristics, especially in terms of age, with adolescent and young adult admissions dropping dramatically [19]. Similar findings were observed in another study conducted in Saudi Arabia in a tertiary hospital, which analyzed the characteristics of admissions since the first reported case of COVID-19; this study showed a decrease in the number of admissions, as well as an increase in age, LOS, and complexity of clinical patients. [20]

The results for the 40 most frequent medical and surgical causes of hospitalization in our hospital show a pattern similar to the one described above, with a decrease in the number of admissions and a change in the patient profile. Our results are consistent with those described in a study in Brazil, which analyzed admissions and mortality during the pandemic crisis, showing a decrease in admissions in most ICD-10 groups [21]. Another study in Alberta, Canada, found a significant decrease in emergency admissions for medical and surgical conditions, with a more marked decrease in admissions for the exacerbation of chronic obstructive pulmonary disease (COPD) and unspecified pneumonia [12].

Regarding the specific profiles of patients hospitalized for medical conditions, we can highlight an increase in the severity of the clinical picture in two pathologies: other types of pneumonia and vaginal delivery. An Italian study in March 2020 also observed an increase in admissions for respiratory diseases and an increased severity [22].

Regarding the profile of patients undergoing surgical procedures, an increase in the severity of patients undergoing “cholecystectomy”; “major pancreas and liver procedures”; “hernias, except inguinal, femoral, and umbilical”; “appendectomy without complicated principal diagnosis”; and “open extracranial vascular procedures” was observed. The PREDICT study, which involved a longitudinal analysis of surgical management during the pandemic in 18 countries, concluded that the first pandemic wave significantly impacted surgical patients, directly and indirectly, by increasing the severity of surgical conditions and the mortality of these patients, regardless of COVID status. One of the reasons mentioned to explain this finding is the redistribution of available surgical staff during the pandemic [23].

For the region of Andalusia, the overall excess mortality during the second pandemic wave was 21.7% [24]. In our setting, a previous study also conducted at the VRUH [6] during the first pandemic wave (March to June 2020), analyzing the clinical pattern and mortality of hospitalized patients, showed a sharp decrease in non-COVID hospitalizations and important changes in the spectrum of admitted patients (increase in the LOS, age, severity, and non-COVID in-hospital mortality), as well as an excess of overall mortality in the health area for which the VRUH is the hospital of reference [6].

The main limitation of our analysis is that it is a single-center study. In addition, this study focuses only on hospital admissions and does not provide results of changes in specialized outpatient care. As for its strengths, it is a real-world, comprehensive analysis considered in the clinical practice, including all the patients hospitalized during the study period and all available epidemiological, demographic, and clinical variables. The comparison with the previous two years shows how the pandemic has changed hospital care.

The present study shows that the impact of the pandemic on the profile and severity of hospitalized patients without COVID remained very significant during the second pandemic wave, although somewhat less than that during the first wave for most of the indicators studied [6]. However, it seems that the experience gained from the management of the first pandemic wave did not lead to an overall meaningful improvement in healthcare management or the outcome variables assessed during the second wave. Indeed, the reduction in non-COVID admissions remained similar in both waves (22.3%) during the periods of excess mortality. The decrease in the complexity level and the increase in the severity level were also very similar. In-hospital excess mortality was the same for both waves (26% on average). Hospital admissions during the second pandemic wave proved to be an independent risk factor for in-hospital mortality, as was also evident for the first wave.

The increase in non-COVID in-hospital mortality in the second half of 2020 compared with previous years is probably related to the higher severity and level of mortality of patients who were eventually admitted to hospitals. The excess indirect mortality due to SARS-CoV-2 may be due to various causes, such as the difficulty and delay in accessing health services, the restrictive measures put in place, or the postponement of non-urgent procedures and interventions; these factors may have led to an increase in the number of patients dying at home and a delayed admission of those coming to hospitals. A study analyzing the mortality in 24 European countries during the period of March to June 2020 and comparing it with the same one during the years 2016–2019 has shown similar results [25].

## 5. Conclusions

In conclusion, this study allows us to understand and quantify the large impact caused by the second pandemic wave in our healthcare setting, which proved to be very similar to that caused by the first wave. There was a change in the clinical profile of patients without COVID, with an increase in age, LOS, and severity, as well as an increase in in-hospital mortality despite the decrease in hospital admissions. This analysis provides important information to help understand the indirect impact of the pandemic on healthcare, inform public health policy development, and guide health management strategies that should be implemented in response to future pandemics or other healthcare crises.

## Figures and Tables

**Table 1:** Characteristics of non-COVID patients admitted to the Virgen del Rocío University Hospital (VRUH), Seville, Spain, between 22 June and 6 December 2020 (Period 1), and during the period of excess mortality (Period 2, including the addition of the sub-periods 23 June 2020 to 26 June 2020, 12 July 2020 to 14 August 2020, and 6 September 2020 to 6 December 2020), compared with the same time periods in 2018–2019.

	Period 1 (Dates Corresponding to the Second Wave of the Pandemic in Seville in 2020, vs. Same Time Period in 2018–2019)			Period 2 (Dates Corresponding to the Period of Excess Mortality in Andalusia during the Second Wave of the Pandemic in 2020, vs. Same Time Period in 2018–2019)		
	2020	2018–2019	*p* Value	2020	2018–2019	*p* Value
Total admissions, *n*	18,260	44,877		11,760	30,292	
Average annual admissions, *n*	18,260	22,439	<0.001	11,760	15,146	<0.001
Sex (male), *n* (%)	8344 (45.7)	21,023 (46.8)	0.008	5333 (45.3)	14,215 (46.9)	0.003
Age, mean ± SD	50.10 ± 24.76	48.77 ± 25.16	<0.001	49.99 ± 24.67	48.99 ± 25.16	<0.001
Emergency admissions, *n* (%)	11,067 (60.60)	24,647 (54.92)	<0.001	7198 (61.2)	16,473 (54.4)	<0.001
Days of hospitalization, mean ± SD	7.02 ± 11.67	6.74 ± 12.14	0.008	7.15 ± 12.42	6.68 ± 11.56	<0.001
Days of hospitalization in ICU, mean ± SD	0.74 ± 6.38	0.70 ± 5.38	0.495	0.73 ± 6.59	0.71 ± 5.48	0.793
Medical APR DRG, *n* (%)	10,947 (60.00)	26,345 (58.7)	0.004	7130 (60.6)	17,553 (57.9)	<0.001
Severity APR-DRG level, mean ± SD	1.79 ± 0.84	1.72 ± 0.79	<0.001	1.80 ± 0.84	1.71 ± 0.79	<0.001
Mortality APR-DRG level, mean ± SD	1.51 ± 0.77	1.44 ± 0.73	<0.001	1.52 ± 0.78	1.44 ± 0.73	<0.001
APR-DRG weight, mean ± SD	0.92 ± 1.13	1.17 ± 1.36	<0.001	0.92 ± 1.12	1.17 ± 1.34	<0.001
APR-DRG-adjusted LOS index, mean ± SD	0.94 ±1.18	0.94 ± 1.31	0.978	0.95 ± 1.35	0.94 ± 1.47	0.440
Deaths at hospital, *n* (%)	899 (4.9)	1753 (3.9)	<0.001	608 (5.2)	1190 (3.9)	<0.001

Data are shown as *n* (%) or mean ± SD; SD, standard deviation; ICU, intensive care unit; APR-DRG all-patient refined diagnosis-related group; LOS, length of stay.

**Table 2:** In-hospital mortality at the VRUH between 22 June and 6 December 2020 (Period 1: the second COVID-19 pandemic wave in Seville), compared with the same period in 2018–2019.

	2020	2019	2018	*p* Value
Admissions in VRUH	19,830	22,499	22,378	<0.001
Non-COVID	18,260	22,499	22,378	
COVID	1570	n/a	n/a	
In-hospital deaths	1165	876	887	
Non-COVID	899	876	887	
COVID	266	n/a	n/a	
In-hospital mortality per 100 admissions	5.9	3.9	3.9	<0.001
Non-COVID	4.9	3.9	3.9	<0.001
COVID	16.9	n/a	n/a	

VRUH: Virgen del Rocío University Hospital, Seville, Spain; n/a, not applicable.

## Data Availability

The data sets used and analyzed during the current study are available from the corresponding author upon reasonable request.

## Supplementary Materials

The supplementary information can be accessed and downloaded at: https://www.mdpi.com/article/10.3390/jcm12175507/s1. The provided content includes Table S1: The characteristics of non-COVID patients admitted for the 40 most common medical APR-DRGs at Virgen del Rocío University Hospital (VRUH), Seville, Spain, from 22 June to 6 December 2020 (Period 1), in comparison with the corresponding time in 2018–2019. Table S2: The characteristics of non-COVID patients admitted for the 40 most prevalent surgical APR-DRGs at VRUH, Seville, Spain, during the same period, together with the matching time intervals in 2018–2019.
